# Effects of visually simulated roll motion on vection and postural stabilization

**DOI:** 10.1186/1743-0003-4-39

**Published:** 2007-10-09

**Authors:** Shigehito Tanahashi, Hiroyasu Ujike, Ryo Kozawa, Kazuhiko Ukai

**Affiliations:** 1 School of Science and Engineering, Waseda University, Tokyo, Japan; 2 Institute for Human Science and Biomedical Engineering, AIST, Tsukuba, Japan; 3 School of Psychology, Chukyo University, Nagoya, Japan

## Abstract

**Background:**

Visual motion often provokes vection (the induced perception of self-motion) and postural movement. Postural movement is known to increase during vection, suggesting the same visual motion signal underlies vection and postural control. However, self-motion does not need to be consciously perceived to influence postural control. Therefore, visual motion itself may affect postural control mechanisms. The purpose of the present study was to investigate the effects of visual motion and vection on postural movements during and after exposure to a visual stimulus motion.

**Methods:**

Eighteen observers completed four experimental conditions, the order of which was counterbalanced across observers. Conditions corresponded to the four possible combinations of rotation direction of the visually simulated roll motion stimulus and the two different visual stimulus patterns. The velocity of the roll motion was held constant in all conditions at 60 deg/s. Observers assumed the standard Romberg stance, and postural movements were measured using a force platform and a head position sensor affixed to a helmet they wore. Observers pressed a button when they perceived vection. Postural responses and psychophysical parameters related to vection were analyzed.

**Results:**

During exposure to the moving stimulus, body sway and head position of all observers moved in the same direction as the stimulus. Moreover, they deviated more during vection perception than no-vection-perception, and during no-vection-perception than no-visual-stimulus-motion. The postural movements also fluctuated more during vection-perception than no-vection-perception, and during no-vection-perception than no-visual-stimulus-motion, both in the left/right and anterior/posterior directions. There was no clear habituation for vection and posture, and no effect of stimulus type.

**Conclusion:**

Our results suggested that visual stimulus motion itself affects postural control, and supported the idea that the same visual motion signal is used for vection and postural control. We speculated that the mechanisms underlying the processing of visual motion signals for postural control and vection perception operate using different thresholds, and that a frame of reference for body orientation perception changed along with vection perception induced further increment of postural sway.

## Background

Virtual Reality (VR) technology has developed rapidly thanks to progress in information technology and computer graphics. The applications of VR technology have expanded to various fields including health and medical services, the amusement industry, architectural design, and others. While VR technology is useful for these applications, it can sometimes have a negative effect, known as "visually induced motion sickness." The cause of this has often been described by sensory conflict or sensory rearrangement theory [1]. To reduce this negative effect of VR technology, we need to understand how visual information is used for perception and the control of self-motion, especially for applications in rehabilitation, health, and medical services [2-4].

Self-motion is perceived and controlled based on information from different senses, including visual, vestibular and proprioceptive [5]. To date, the literature has largely focused on the role of visual information [6-9], partly because visual information plays a principal role in the perception of self-motion. In fact, motion in a large visual field often induces the perception of self-motion; an effect is known as vection [10,11] in an individual who remains stationary. Visual information also plays major role in postural control. Motion in a large visual field has been shown to increase postural sway involuntarily [7,8,12], and restricting the visual field often destabilizes the body [13]. In the present study, our concern, here, was to whether the visual information contributing to vection is the same as that involved in postural control.

Previous research has demonstrated that vection and postural movement are correlated when visual stimulus motion, which is motion presented in the visual field, was presented. Inclination of the body, defined as postural sway in the present study, was reported to increase during periods of vection as compared to periods of no-vection [14-16]. Wolsley et al. [14] and Thurrell et al. [15] reported that the visually evoked postural response increased during periods of vection, and its direction tended to align with the plane of motion of the visual stimulus. Kuno et al. [16] reported that the magnitude of vection induced by an optokinetic stimulus moving in depth was correlated with the velocity of the stimulus, which in turn was correlated with the magnitude of postural sway in anterior/posterior direction. Moreover, small fluctuations in body movements, defined as postural instability in the present study, were reported to increase with vection. Fushiki et al. [17] reported that vection induced by vertical visual stimulus motion was a significant factor in postural instability in the anterior/posterior direction. Taken together, these findings suggest that the same visual motion signal, which is processed in the visual system, underlies vection and postural control. However, self-motion does not need to be consciously perceived to influence postural control [18]. In fact, Previc and Mullen [18] and Clément et al. [19] have noted that onset latencies of postural change is shorter than that of vection.

The magnitude of postural movements was also reported to increase during visual stimulus motion as compared to conditions with no visual stimulus motion. van Asten et al. [20] reported that a visually simulated rotation induced postural movements involving rotations in the ankle joint. However, this research did not clearly describe whether the postural movements were induced by the visual rotation itself or by vection produced by the visual rotation. If the visual rotation in itself increased postural movements, visual motion affected postural control mechanisms regardless of vection. While the same visual information seems to be used for vection and postural control, we need to make clear whether visual motion in itself affects postural control mechanisms.

The goal of the present study was to investigate the effect of visual motion and vection on postural control mechanisms. To this end, we measured and compared postural movements in terms of the center of foot pressure and head position during three different periods: no-visual-stimulus-motion, visual-stimulus-motion without vection, and visual-stimulus-motion with vection. To investigate the effect in detail, we analyzed the postural movement data by postural sway and postural instability in both the left/right and anterior/posterior directions.

## Methods

### Observers

Eighteen adults, aged 19–72 years (13 females and 5 males; 39.7 ± 14.9 years), were recruited from local residents in the Tsukuba city area. All observers participated in the study after giving their informed written consent in accordance with the provision of AIST, (National Institute of Advanced Industrial Science and Technology), ergonomics experiment policy, and were free to withdraw at any time during the experiment. The experimental protocol was approved in advance by the Institutional Review Board of AIST. The observers were naïve as to the purpose of the experiment, and had normal, or corrected-to-normal, visual acuity by testing with Randolt's test chart at 5 m and optometer, and no history of optic nerve disease.

### Stimulus and Apparatus

**Figure 1:**
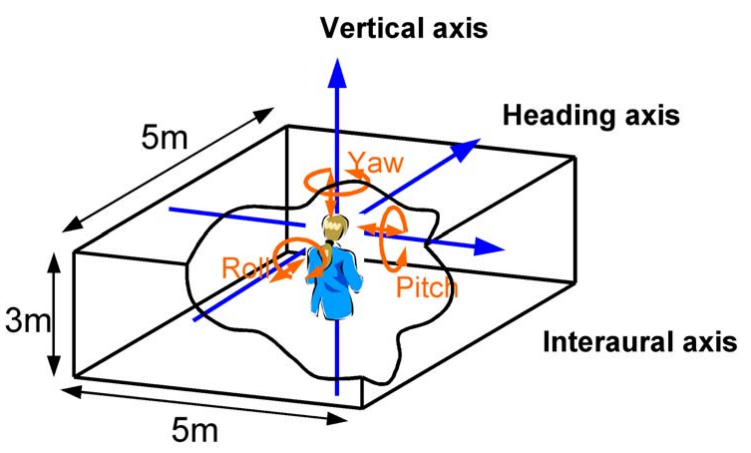
**Schematic illustration of the virtual environment**. Observers stood at the center of the rectangular space whose wall was textured with one of the two different patterns shown in Figure 2.

**Figure 2:**
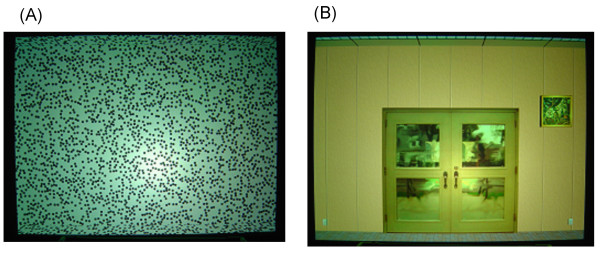
**The two different visual contexts**. Textures presented on a wall of the rectangular solid were either (a) a random-dot texture, or (b) a CG-image that simulated an ordinary room.

A moving visual image virtually simulated rotation along the roll axis. As shown in Figure 1, the observer was located at the center of the virtually simulated rectangular space that was 5 × 5 × 3 m (width × depth × height). Two different visual contexts were produced on the inside walls of the rectangular space (Figure 2). One was a random-dot pattern consisting of black dots (2.29 cd/m^2^) on white walls (43.6 cd/m^2^), and the other was a pattern that simulated an ordinary room (46.2 cd/m^2 ^for a typical wall). The luminance values indicated were measured for the central 20 deg, and those in the periphery decreased to 31% of the central value due to the characteristics of the back-projection system described below. Despite the luminance difference across the screen, the appearance of images differed very little between the center and the periphery. The diameter of each dot of the random-dot pattern was 4 cm on the wall, and the density of the dot area on the wall was 22%. The pattern simulated an ordinary room including a double door, windows, yellowish-brown wall, linoleum-covered floor, and ceiling with area lighting.

The visual images were created online on a Windows-based PC (Pentium 4, 2.0 GHz) with OpenGL, and were back-projected on a screen with LC projector (EPSON, ELP-7700). The frame rate was 60 Hz. The image size was 1024 × 768 pixels (0.3 mm/pixel), or 82 × 67 deg from a viewing distance of 1 m (an appropriate distance for viewing the stimulus produced with perspective projection). The height of the projected area of the visual image on the screen was adjustable to the vantage point of each observer in the standing position so that visual scene was horizontal. The experimental room was light-proofed; no lights other than the projector were on during the experiment.

Observers' postural movements were measured with two different parameters: center of foot pressure (COP) and head position. First, the COP was measured by a force platform system (Kyowa Electronic Instruments, M98-6188), which recorded pressure data for four different points on the platform at 100 Hz using strain gauges. The data underwent a 12 bit AD conversion. Based on the digitized data, the COP was calculated; the maximum error of the COP was within ± 1.63 mm over 30 kg of body weight. Second, head position was measured in six degrees of freedom (the rotations and translations along the axes of yaw, pitch and roll of the standing observer) by an electromagnetic tracking system (Polhemus 3 space Fastrack). The transmitter for the system was positioned just above the observer's head, 218 cm above the surface of the force platform. The receiver for the system was attached on the top of a helmet worn by the observer. The head position data were measured and recorded at 30 Hz, with a spatial resolution of 0.025 deg (0.0002 inches) and a time delay from position changes to detection of 4.0 ms.

### Procedure

Before starting the experimental trials, we measured the height of each observer's eye position to adjust the height of the visual image on the screen. Then, observers were dark adapted with eye masks in the dark experimental room for 10 minutes. Each observer carried out four trials, corresponding to each of the combinations of visual rotation directions (clockwise/counterclockwise, or CW/CCW) along the roll axis and the two different stimuli: the random-dot or CG image stimulus. The order of the combinations was counterbalanced across observers.

At the beginning of each trial, the observer stood on the force platform and their baseline COP and head position were recorded. Each trial started with stationary image for 10 s, and then the moving image was presented for 120 s, followed by the stationary image for 60 s. The observer was instructed to assume the standard Romberg stance on the force platform and to passively look at the stimulus in the standing position. Whenever the observer perceived self-motion (i.e., vection), they pressed a button held in their left hand. Button presses were recorded at 60 Hz. After the trial, the observer reported vection strength over the trial on an 11 point scale with 0 representing "no vection was perceived," and 10 representing "vection so strong that the perceived self-motion could not be differentiated from real physical motion."

### Data analysis

Experimental data obtained from three out of the 18 observers were discarded, resulting in 15 observers (11 females and 4 males; 36.9 ± 12.8 years). Two of these observers did not perceive vection in all trials or they only perceived vection in one trial. The third observer whose data was discarded often held the safety bar when watching the stimulus to avoid falling down; he strongly perceived vection in all trials.

We re-sampled the COP and head position data at 60 Hz to compare these data across different periods, which were categorized based on the subjective responses of vection or no-vection. Because the data were originally sampled at different rates for different parameters, (the COP at 100 Hz, the head position at 30 Hz and the subjective response at 60 Hz), the re-sampled data at 60 Hz were obtained by weighted averaging of the two adjacent originally sampled data points for each parameter. Moreover, the COP and head position data were decomposed into those in the left/right (L/R) and anterior/posterior (A/P) directions, to look separately at the data parallel and orthogonal to the stimulus projection surface.

The data were examined both within the trials and across the trials. First, the data were examined within each trial to investigate the effects of visual-stimulus-motion and vection on postural movement, and the after-effects. To investigate the effects of visual-stimulus-motion and vection, we separated the COP and head position data into two different pairs of periods: one, visual-stimulus-motion versus no-visual-stimulus-motion, and two, vection versus no-vection. Here, the visual-stimulus-motion represents motion of visual image, while no-visual-stimulus-motion represents no motion of visual image. The period of no-visual-stimulus-motion corresponded to the first 10 s of each trial, in which the visual stimulus was stationary. To investigate the after-effects, we looked at the COP and head position data obtained after the end of the visual-stimulus-motion. We calculated averages for 10 s periods starting from just after the end of the visual-stimulus-motion, and the averages were continuing with shifting the 10 s period every 1/60 s until the end of the period reached to the end of the trial.

Second, the data were examined across the trials to investigate the effects of repeated exposure to the stimulus on vection and postural movement. We compared four different psychophysical parameters of vection across the trials: (1) onset latency of circular-vection (i.e., the time elapsed between the onset of optokinetic stimulation and the first subjective report of perceived self-motion), (2) the number of vection episodes, (3) the total time spent perceiving circular-vection within the 120 s period of visual-stimulus-motion in a trial, and (4) the observer's ratings of vection strength.

In the analyses described above, we examined averages of the COP and head position data across all the trials and all the observers. Before taking the averages across all the trials, we made bias corrections for the COP and head position data within each trial by subtracting average values of the COP or head position during the initial no-visual-stimulus-motion period from the original values. Then, the sign of values in the L/R direction for trials in which the stimulus rotated in the CCW direction was inverted. All the averaged data described below were calculated using this process unless otherwise specified. Positive values for the COP and head position data in the L/R direction indicate that the sway occurred toward the right. Positive values in the A/P direction indicate that the sway occurred as forward motion. The statistics appeared will be t-test, otherwise specified.

## Results

### Postural response in rotation

**Figure 3:**
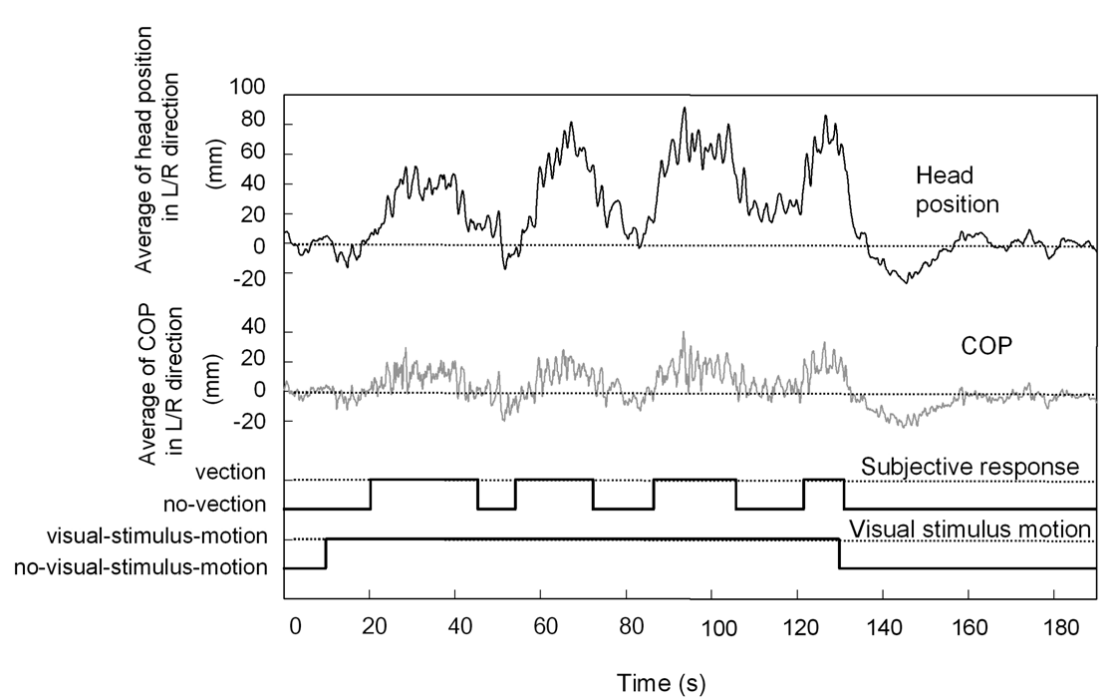
**Sample data for head position, COP, and vection responses in a typical trial**. The data labeled as motion indicates the period of visual-stimulus-motion, while the data labeled as no-motion indicates the periods of no-visual-stimulus-motion. The positive vertical values indicate that head position and COP changes were in the direction of the visual-stimulus-motion. The value zero in the ordinate represents the average value during no-visual-stimulus-motion, prior to any visual-stimulus-motion.

During stimulus presentation, body sway and head position changed in the same direction as the visual stimulus rotation for all observers. That is, the observer's body inclined rightward when the stimulus rotated in a clockwise direction. Moreover, postural instability of the COP and head position changes also occurred during stimulus presentation. This is shown in Figure 3, which illustrates the typical data for COP and head position in a single trial for one observer during rightward of visual roll motion. To look at these changes of postural sway and postural instability in detail, we examined the COP and head position data in terms of average position and fluctuation of positions, and compared each of them between different periods: visual-stimulus-motion versus no-visual-stimulus-motion, and vection versus no-vection. The average positions were computed for each COP and head position as arithmetic averages across periods of the identical condition (visual-stimulus-motion or no-visual-stimulus-motion) or of the same category of perception (vection or no-vection). The fluctuations were analyzed as averaged standard deviation, or averaged-SD, that was computed for each COP and head position as arithmetic averages of standard deviations across periods of the identical condition or of the same category of perception.

**Figure 4:**
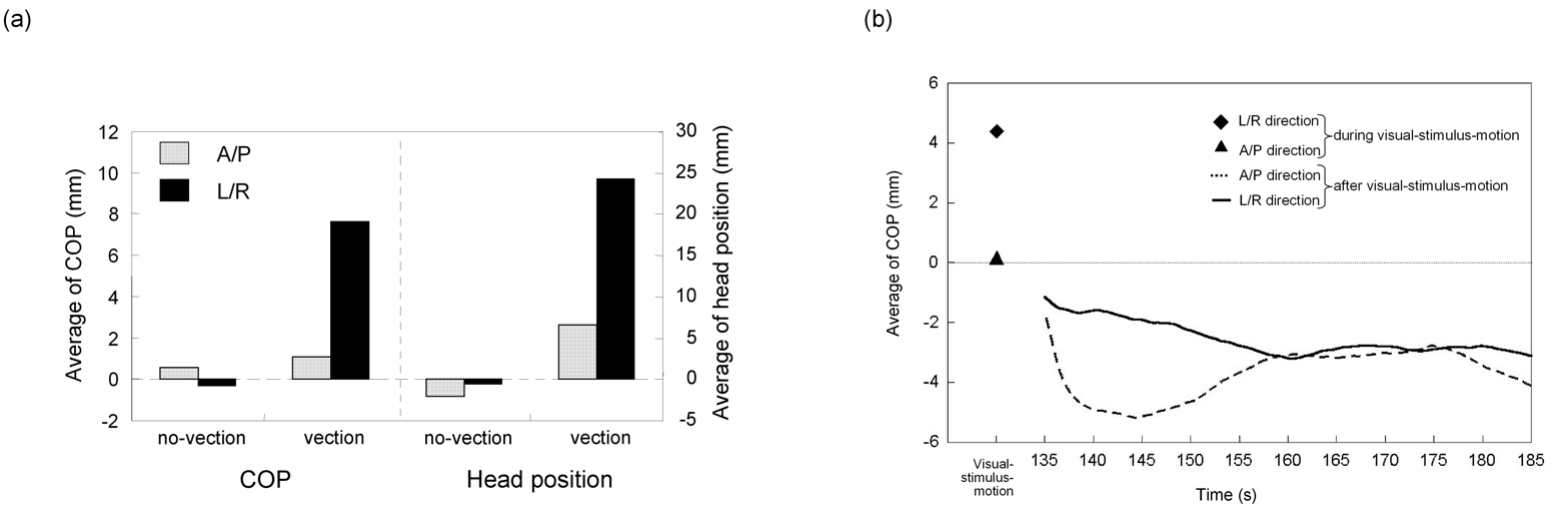
**Mean postural responses for the L/R and A/P directions**. (a) Mean of COP or head position during vection and no-vection in both the L/R and A/P directions. Also shown are the continuous mean values of mean of either (b) COP or (c) head position after the visual stimulus motion ceased. The two data points in the left-most part of (b) and (c) represent averaged values in the L/R and A/P directions during visual-stimulus-motion.

The postural movement represented by COP and head position clearly deviated in the direction of visual stimulus rotation, and this was more pronounced during periods of vection. The average COPs during vection and no-vection are shown in Figure 4a, with positive values of COP matching the direction of the visual stimulus rotation. The average COP in the L/R direction during vection was significantly greater than that during no-vection (*p *< 0.001). In contrast, the average COP in the A/P direction during vection did not differ significantly from that during no-vection (*p *> 0.1). The average head positions during vection and no-vection are also shown in Figure 4a. Consistent with the COP results, the average head position in the L/R direction during vection was significantly greater than that during no-vection (*p *< 0.01), and the average head position in the A/P direction did not differ significantly between vection and no-vection (*p *> 0.1). Moreover, the average COP and head position in the L/R direction were significantly greater during visual-stimulus-motion than during no- visual-stimulus-motion (for COP, *p *< 0.01; for head position, *p *< 0.001). No significant differences were seen for the motion/no-motion comparisons in the A/P direction for COP and head position (*p *> 0.1).

**Figure 5:**
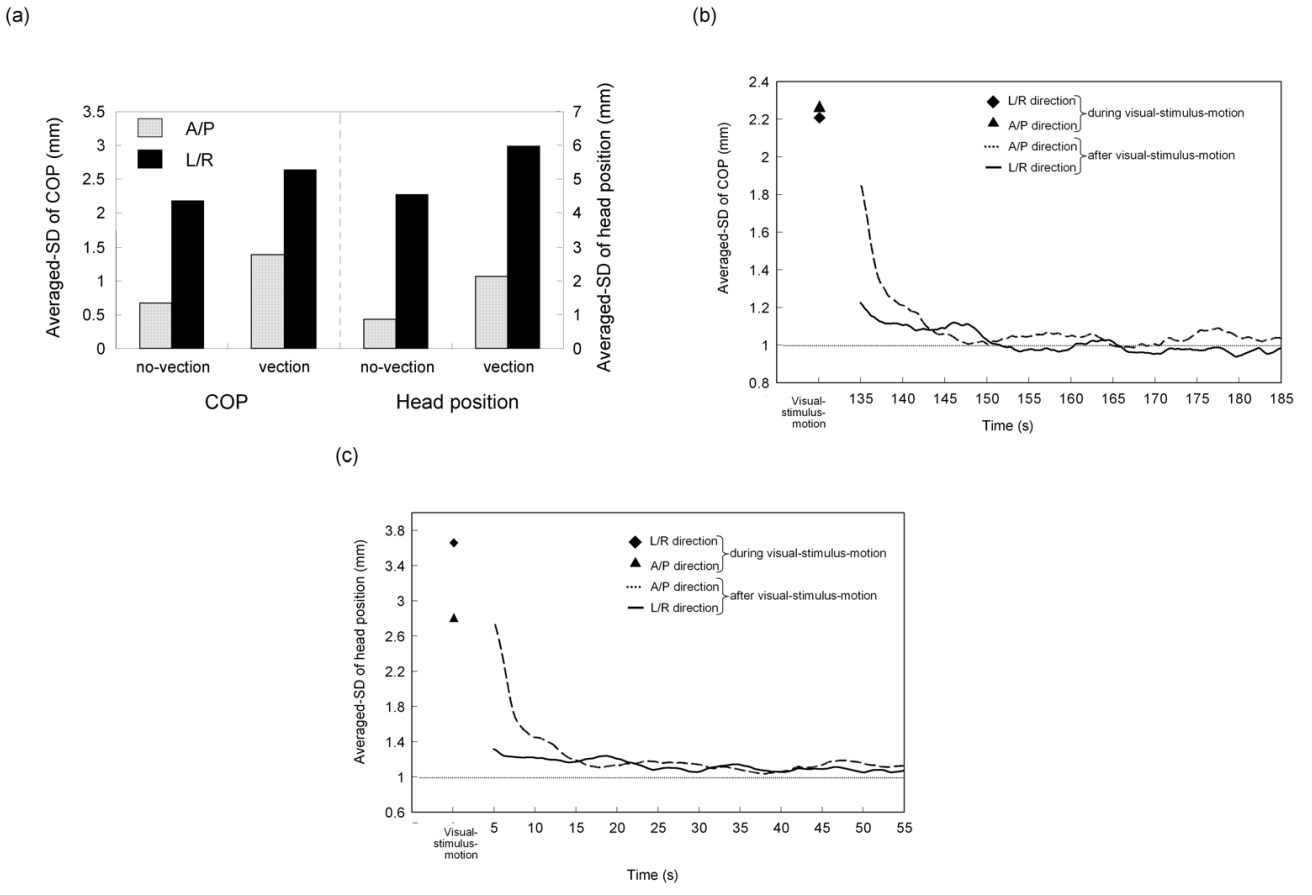
**Averaged-SD of postural responses for L/R and A/P directions**. (a) Averaged-SD of COP or head position during vection and no-vection in both the L/R and A/P directions. Also shown are the continuous values of averaged-SD of either (b) COP or (c) head position after the visual stimulus motion ceased. The two data points in the left-most part of (b) and (c) represent averaged-SD in the L/R and A/P directions during visual-stimulus-motion.

Postural movements clearly fluctuated in the L/R and A/P directions, and this was more pronounced during periods of vection. As shown in Figures 5a, the averaged-SD of COP in both the L/R and A/P directions during vection were significantly greater than those during no-vection (*p *< 0.05). Figure 5a depicted also the averaged-SD of head position during vection and no-vection. The averaged-SD of head position in both the L/R and A/P directions during vection were significantly greater than those during no-vection (for L/R, *p *< 0.01; for A/P, *p *< 0.05). Moreover, the averaged-SD of COP and head position in both the L/R and A/P directions during visual-stimulus-motion were significantly greater than those during no-visual-stimulus-motion (*p *< 0.001). In addition, for COP in motion and head position in no-motion, the averaged-SD did not differ significantly between the L/R and A/P directions (*p *> 0.1). Similarly, the averaged-SD of COP and head position in both the no-vection and the vection periods did not differ between the L/R and A/P directions (*p *> 0.1).

### Postural response after rotation ceased

**Figure 6:**
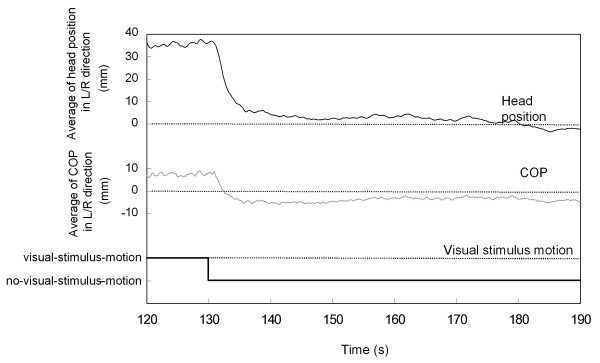
**Averaged head position and COP for latter part of experimental trial**. The data labeled as motion indicates the period of visual-stimulus-motion, while the data labeled as no-motion indicates the periods of no-visual-stimulus-motion.

Immediately following the end of the visual stimulus rotation, COP in the L/R direction changed drastically. The values for COP and head position decreased steeply immediately after the visual-stimulus-motion ended (130 s into the trial), as illustrated in Figure 6, in which the COP and head position recordings averaged across all trials for all 15 observers are shown from 120 s after the trial started to the end of the trial. Moreover, COP decreased to a negative value, indicating that COP was inclined in the opposite direction relative to the postural sway that occurred during visual-stimulus-motion. Although head position also switched to the opposite direction during visual-stimulus-motion, it did not reach a negative value for the duration of the after-motion period, as shown in Figure 6.

The COP and head position data in terms of average position and averaged-SD were subsequently compared across different periods: no-vection versus vection during visual stimulus rotation, as well as across successive 10 s periods starting from just after the end of the visual-stimulus-motion and continuing every 1/60 s until the end of 10 s period reached to the end of the trial. The values were arithmetically averaged across all the trials for all the observers in each of the L/R and A/P directions, and are shown in Tables 1, 2, 3 and 4 along with the standard deviations. The detailed time-series data are shown graphically in Figures 4c and 4d, and 5c and 5d. The values were calculated by taking average and standard deviation, or SD, of the data sampled at 60 Hz for 10 s periods starting the end of the visual stimulus rotation and continuing until the end of 10 s period reached to the end of the trial. The average and SD values were further averaged across all the trials for all the observers in each of the L/R and A/P directions. Those average values are plotted at the middle of the period from when the values were averaged; for example, the COP value averaged between the periods of 130 and 140 s is plotted at 135 s along the abscissa.

**Table 1:** Averages of center of foot pressure during and after visual-stimulus-motion

period^*1^	L/R		A/P	
	average	SD	average	SD
no-vection	1.10	6.84	0.59	7.36
vection	7.65	6.74	-0.30	8.69
130 to 140s	-1.66	5.96	-0.55	11.26
140 to 150s	-5.31	6.51	-0.38	10.48
150 to 160s	-3.67	5.52	-1.11	9.44
160 to 170s	-3.17	5.24	-1.59	8.80
170 to 180s	-2.79	5.57	-0.18	8.47
180 to 190s	-4.11	5.59	0.95	8.46

^*1^: The numbers represent the time (s) after the trial started.

**Table 2:** Averages of head position during and after visual-stimulus-motion

period^*1^	L/R		A/P	
	average	SD	average	SD
no-vection	7.86	22.67	-2.42	14.70
vection	29.13	24.50	-0.66	19.08
130 to 140s	13.84	20.25	0.69	24.04
140 to 150s	3.02	16.26	0.22	19.57
150 to 160s	2.97	13.13	1.12	17.60
160 to 170s	2.54	10.75	1.65	16.58
170 to 180s	1.48	11.46	-1.01	15.46
180 to 190s	-2.07	11.29	-2.42	14.31

^*1^: The numbers represent the time (s) after the trial started.

**Table 3:** Averaged standard deviation of center of foot pressure during and after visual-stimulus-motion

period^*1^	L/R		A/P	
	average	SD	average	SD
no-vection	1.40	0.57	1.15	0.87
vection	1.81	0.86	1.69	0.84
130 to 140s	1.85	1.08	1.22	0.72
140 to 150s	1.06	0.51	1.09	0.65
150 to 160s	1.05	0.45	0.97	0.53
160 to 170s	1.00	0.49	1.02	0.61
170 to 180s	1.07	0.48	0.97	0.65
180 to 190s	1.04	0.40	0.98	0.50

^*1^: The numbers represent the time (s) after the trial started.

**Table 4:** Averaged standard deviation of head position during and after visual-stimulus-motion

period^*1^	L/R		A/P	
	average	SD	average	SD
no-vection	1.46	0.96	1.10	1.33
vection	2.26	2.03	1.85	1.37
130 to 140s	2.76	2.44	1.31	1.05
140 to 150s	1.20	0.86	1.17	0.88
150 to 160s	1.17	0.75	1.08	0.66
160 to 170s	1.09	0.84	1.14	0.80
170 to 180s	1.14	0.78	1.09	0.82
180 to 190s	1.13	0.68	1.07	0.69

^*1^: The numbers represent the time (s) after the trial started.

The detailed analysis confirmed our above findings regarding the postural movement immediately after the cessation of the visual stimulus rotation. Using t-tests, we compared the average COP or head position values for the 10 s period from 140 to 150 s with those during the 10 s no-visual-stimulus-motion period prior to visual stimulus rotation. We found that the average COP in the L/R direction steeply decreased to a negative value during the 140 to 150 s period as compared to the pre-motion value (*p *< 0.01). In contrast, the average COP in the A/P direction and the average head position in the L/R and A/P direction essentially returned to their initial position prior to the visual-stimulus-motion, showing no significant differences pre- and post-motion for A/P COP, L/R head position, and A/P head position (*p *> 0.1). Averaged-SD for both measures in both directions decreased back to their values shown during the initial no-motion period. As a result, no significant differences were seen for pre- and post-visual-stimulus-motion comparisons for L/R COP, A/P COP, L/R head position, and A/P head position (*p *> 0.1).

### Across trial differences

**Figure 7:**
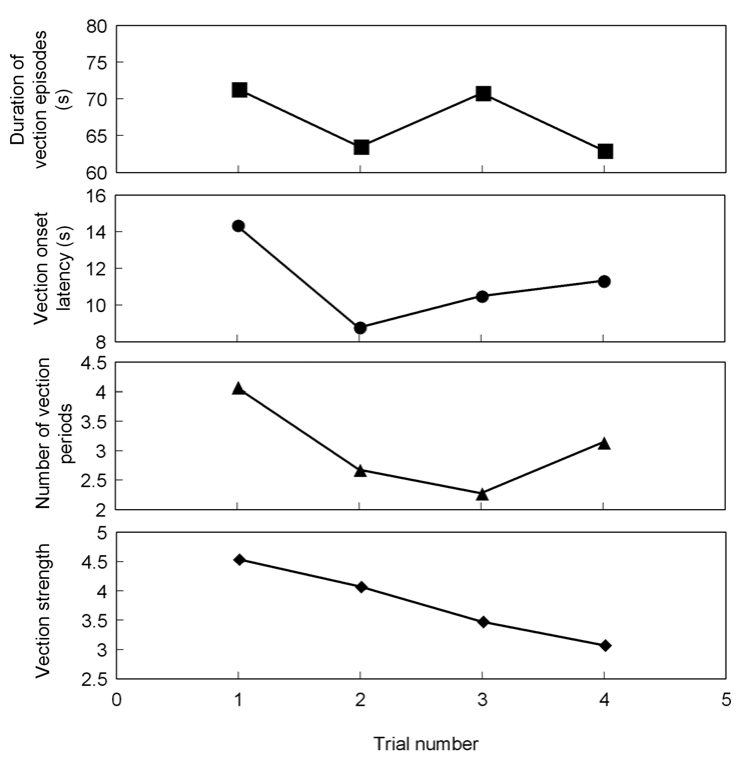
**Vection parameters across different trials**. Means of the psychophysical parameters of circular vection measured during the perception of vection for each trial.

Two of the four different psychophysical parameters of vection (the number of vection episodes and vection strength) differed slightly across the four trials, as shown in Figure 7. Analysis of variance showed the effect of trial was significant for the number of vection episodes and vection strength (*p *< 0.01). However, there was no effect of trial for vection onset latency and the duration of vection episodes (*p > *0.1). For the number of vection episodes and vection strength, however, the results were not significant when corrected for multiple comparisons.

**Figure 8:**
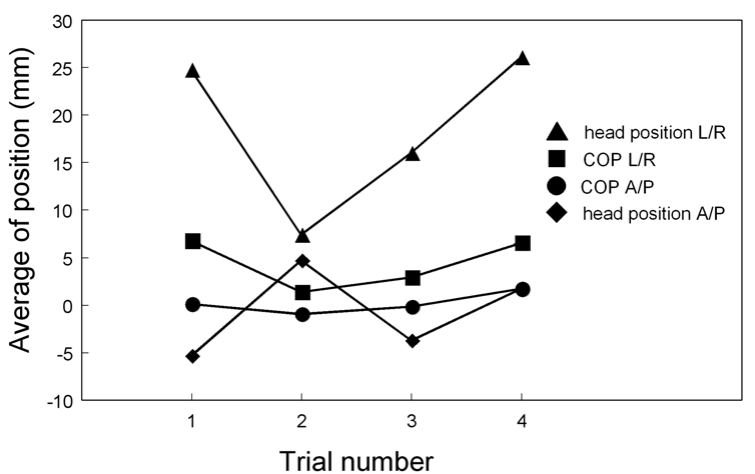
**Postural sway in both L/R and A/P directions across different trials**. Means of the postural responses measured during visual-stimulus-motion.

As shown in Figure 8, the COP and head position in the L/R direction differed slightly across the four trials during visual-stimulus-motion. Analysis of variance showed the effect of the trial was significant in the L/R direction for both COP (*p *< 0.01) and head position (*p *< 0.05). No significant effects were seen for the equivalent across trial comparisons in the A/P direction for COP and head position (*p *> 0.1).

### Stimulus differences

When the two visual stimuli were compared, no significant differences were seen across the various psychophysical parameters of vection, or for the average position and fluctuation of postural movement (for all *t*-tests, *p *> 0.1).

## Discussion

Our results suggested that visual motion in itself affects postural control. Postural sway, measured in the present study in terms of COP and head position, was significantly larger during visual-stimulus-motion without vection than during no-visual-stimulus-motion. Postural sway during visual-stimulus-motion without vection, however, was significantly smaller than periods of visual-stimulus-motion with vection, consistent with previous studies [14-16]. These results suggest that the increasing postural sway seen with visual-stimulus-motion is not simply attributable to vection induced by visually simulated rotation.

Postural sway during visual-stimulus-motion without vection showed two definite characteristics. First, the direction of postural sway was parallel to the plane of the visually presented roll motion, consistent with previous findings during visual-stimulus-motion with vection [14,15]. That is, postural sway occurred in the L/R direction, but not in the A/P direction, when the visually simulated roll motion was presented in the frontoparallel plane. Second, postural sway in the L/R direction consistently corresponded to the direction of the visually simulated roll motion. For example, observers inclined rightward when the stimulus rotated in the clockwise direction. These two characteristics found for postural sway during visual-stimulus-motion without vection are consistent with those reported previously for postural sway during vection [14,15], and these characteristics during vection were replicated in the present study. Thus, the direction of postural sway was parallel to that of the visually presented roll motion that induced vection, and postural sway in the L/R direction consistently corresponded to the direction of visual rotation that induced vection. These common characteristics indicated that visual motion signals were processed in the same manner, whether or not they induced vection.

The results demonstrating an increment in postural instability, or small fluctuations of body movements, during visual-stimulus-motion may be most consistent with a postural control mechanism that does not rely upon visual information. In the present study, postural instability significantly increased during visual rotation, and further increased during vection perception. Because of this increment during vection, it can be concluded that vection itself affects postural instability. Also of note, in contrast to postural sway, postural instability increased not only in the L/R direction but also in the A/P direction during the presentation of a visually simulated roll motion stimulus, and the increase did not differ significant between the two directions. Duarte and Zatsiorsky [21] reported that variability of COP displacement, equivalent to postural instability as described here, increased when participants occupied leaning postures. Moreover, they showed that the variability of COP displacement increased isotropically in all horizontal directions. This result resembles the present findings of postural instability increasing in both the L/R and A/P directions. Of primary importance, they found that the isotropic increment of the COP area occurred irrespective of whether visual information was or was not available and proposed that this finding may have been due to changes in the pressure distribution on the soles of the feet in leaning positions. Such changes would modify the tactile information available to postural control mechanisms and diminish the usefulness of the information. Because the present results showing increased postural instability during vection perception resemble the results described above during leaning, a similar explanation involving changes in the pressure distribution on the soles of the feet may underlie the findings. Moreover, the relative increment of postural instability during vection as compared to no vection may be induced by postural sway caused by the vection, but not induced by vection in itself.

**Figure 9:**
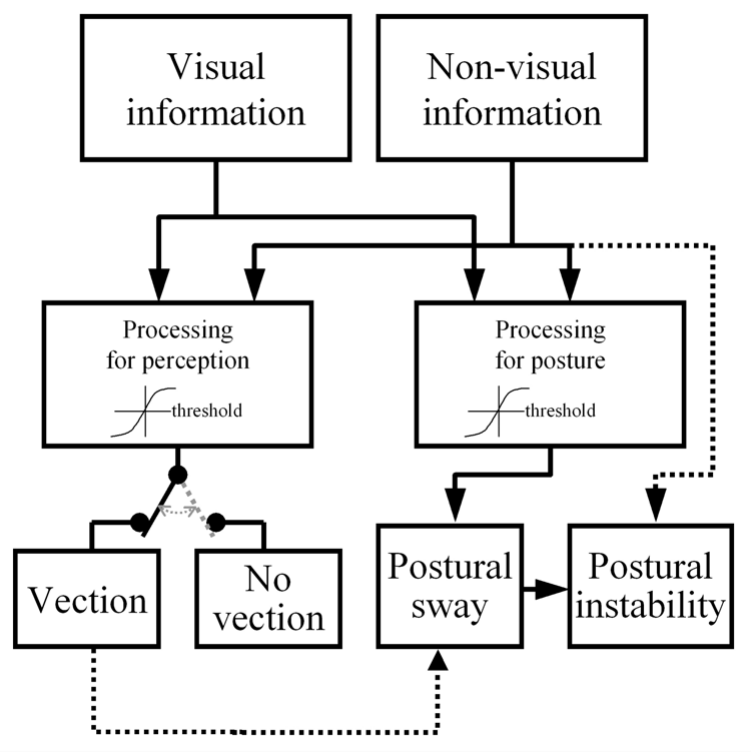
**A model of the relationship between postural control and vection**. Visual and non-visual signals are used for both vection and postural control mechanisms.

The result that visual-stimulus-motion inducing postural sway did not necessarily induce vection may be explained by different thresholds of processing visual motion signals for postural control as compared to vection perception mechanisms. Our results, together with that of previous study [15], suggested that both mechanisms use the same visual information. However, postural sway was even larger during visual-stimulus-motion with no-vection perception than when there was no-visual-stimulus-motion. Therefore, thresholds for postural control and vection mechanisms for processing visual information may be different. This was previously suggested by Previc and Mullen [18] in their discussion of the reasons underlying the different latencies for postural sway and vection. Based on the present results, we developed a schematic diagram illustrating the processes underlying postural control and vection. As shown in Figure 9, both visual and non-visual signals, such as vestibular and somatosensory information about body orientation, are used for postural control and vection mechanisms. The mechanisms weight the signals; if the visual signal exceeds the threshold, postural sway and vection will occur. Strictly speaking, we cannot be certain whether the weights of visual signals in the two mechanisms are different, or the thresholds are different, or both. In the model, postural instability is determined by postural sway (affected by visual and non-visual information) and directly by non-visual information.

In contrast to Previc and Mullen's and Clément et al.'s and reports [18,19] focusing on latencies, our study showed the different effects of visual rotation on postural control between alternations of perceived vection and no perceived vection. Postural sway was significantly larger when vection was perceived as compared to when no vection was perceived. As per the proposed model in Figure 9, the increment of postural sway during vection occurred at suprathreshold level. One possible explanation for this increment may be that the frame of reference for body orientation was shifted when vection was perceived. This shift in the perception mechanism would have also affected postural sway. As there has been considerable discussion on frames of reference, including the proposition that visual perception and postural control have different frames of reference [22,23], investigation into the relation between the type of results shown here and different frames of reference is warranted in future.

After the visual-stimulus-motion stopped, the COP and head position in the L/R direction did not move in the same manner. While the averaged COP inclined in the opposite direction relative to the postural sway that occurred during visual-stimulus-motion, the averaged head position simply returned to the initial position seen prior to visual-stimulus-motion. This may be related to the larger effects of vection on head movements than on body movements, as suggested by Mizuno et al. [24]. During visual-stimulus-motion, the average head position inclined more than the body, or COP, as shown in Figure 3a, presumably because of the effects of vection. When the visual-stimulus-motion ceased, the averaged body position tended to move back to the upright position, as previously shown in ankle muscle stiffness [25]. However, at that time the head was still inclined relative to the body in the same direction as the postural sway that occurred during visual-stimulus-motion. The reason for this is not clear, and the motion after-effect reported by most of the observers upon cessation of the visual-stimulus-motion might have been weak to induce vection that could move the head back to straight position relative to the body. Because of the relative inclination of the head relative to the body, the body, or the COP, might have had to incline beyond the upright position.

One finding that remains to be explained is the lack of any effect of stimulus type. The results did not show a difference in vection and postural sway between the random-dot pattern and the CG-image simulating a room. The information included in the CG-image, such as gravitational direction, horizontal/vertical lines and familiar objects, was expected to provide particularly strong cues for perceived self-rotation. However, the additional cues may have been ineffective in our stimulus situation because the visually simulated roll motion was constant and continuous for both visual contexts.

Although we did not find clear evidence for habituation of postural sway in our experimental paradigm, we did identify some across-trials differences for two of the psychophysical parameters of vection (the number of vection episodes and vection strength), as well as across-trial differences in the average COP and head position in the L/R direction. The other two psychophysical parameters concerning vection (vection onset latency and the duration of vection episodes) did not differ across trials. While the cause of the discrepancy across the different vection parameters is not certain, we speculate that the experimental paradigm did not induce a large degree of habituation.

VR is increasingly been applied as a rehabilitation tool [26]. One problem with this is that rehabilitants in a VR system sometimes suffer from motion sickness [27]. Because motion sickness is often correlated with vection, based on our results it appears that conditions that readily cause motion sickness may also increase the occurrence of increased postural sway and falling. Therefore, it is important to determine which conditions most readily induce vection and motion sickness so that these effects can be countered.
